# Irbesartan in experimental diabetic nephropathy

**DOI:** 10.4103/0253-7613.66849

**Published:** 2010-06

**Authors:** Rajiv Mahajan, Kapil Gupta, Navyug Raj Singh

**Affiliations:** Department of Pharmacology, Adesh Institute of Medical Sciences and Research, Bathinda - 141 109, Punjab, India; 1 Department of Biochemistry, Adesh Institute of Medical Sciences and Research, Bathinda - 141 109, Punjab, India; 2 Department of Pharmacology, Government Medical College, Amritsar - 143 001, Punjab, India

Sir,

We read the article “Biochemical effects of irbesartan in experimental diabetic nephropathy” published in IJP[1] with great enthusiasm. We have the following observations to make regarding this article:

1. This study discussed the beneficial effects of irbesartan in counteracting some biochemical or functional changes of diabetic nephropathy (DN) in experimental animals, while irbesartan has already been approved for use in DN in humans.[2] The relevance of this study is, therefore, debatable.
2. In our opinion, instead of insulin, a drug chosen from the same group or an angiotensin-converting enzyme (ACE) inhibitor having an established role in DN would have been better.
3. In methods, while discussing groups, animals pre-treated with irbesartan and insulin have been referred to as group III and group IV, respectively. However, in all three tables, animals pre-treated with insulin have been referred to as group III and animals pre-treated with irbesartan have been referred to as group IV. Now, which one of these statements is true and figures mentioned in tables belong to which group actually?
4. In results, it is mentioned that insulin pre-treatment completely prevented proteinuria, but proteinuria actually increased with insulin pre-treatment (group III) as compared to diabetic rats (group IV) by 50–60 times. Which one of the statements is true?
5. It is clear that creatinine clearance was 1.1 ± 0.18 ml/min at 0 weeks in normal rats (group I), which suddenly increased to 20.8 ± 0.84 ml/min at 4 weeks. It remained static, but again had a sudden fall from 21.3 ± 0.86 ml/min to 1.2 ± 0.06 ml/min within a period of 4 weeks. Is there any possible explanation why this happened in normal rats?
